# Sequestosome 1 Deficiency Delays, but Does Not Prevent Brain Damage Formation Following Acute Brain Injury in Adult Mice

**DOI:** 10.3389/fnins.2017.00678

**Published:** 2017-12-19

**Authors:** Anne Sebastiani, Christina Gölz, Philipp G. Sebastiani, Wiesia Bobkiewicz, Christian Behl, Thomas Mittmann, Serge C. Thal, Kristin Engelhard

**Affiliations:** ^1^Department of Anesthesiology, University Medical Center of the Johannes Gutenberg University, Mainz, Germany; ^2^Institute of Physiological Chemistry and Pathobiochemistry, University Medical Center of the Johannes Gutenberg University, Mainz, Germany; ^3^Institute of Physiology, University Medical Center of the Johannes Gutenberg University, Mainz, Germany

**Keywords:** traumatic brain injury, protein degradation, autophagy, SQSTM1, p62, BAG3, mouse model

## Abstract

Neuronal degeneration following traumatic brain injury (TBI) leads to intracellular accumulation of dysfunctional proteins and organelles. Autophagy may serve to facilitate degradation to overcome protein debris load and therefore be an important pro-survival factor. On the contrary, clearing may serve as pro-death factor by removal of essential or required proteins involved in pro-survival cascades. Sequestosome 1 (SQSTM1/p62) is a main regulator of the autophagic pathway that directs ubiquinated cargoes to autophagosomes for degradation. We show that SQSTM1 protein levels are suppressed 24 h and by trend 5 days after trauma. In line with these data the expression of *Sqstm1* mRNA is reduced by 30% at day 3 after and stays depressed until day 5 after injury, indicating an impaired autophagy post controlled cortical impact (CCI). To determine the potential role of SQSTM1-dependent autophagy after TBI, mice lacking SQSTM1 (SQSTM1-KO) and littermates (WT) were subjected to CCI and brain lesion volume was determined 24 h and 5 days after insult. Lesion volume is 17% smaller at 24 h and immunoblotting reveals a reduction by trend of cell death marker αII-spectrin cleavage. But there is no effect on brain damage and cell death markers 5 days after trauma in SQSTM1-KO compared with WT. In line with these data neurofunctional testing does not reveal any differences. Additionally, gene expression of inflammatory (*Tnf-*α*, iNos, Il-6*, and *Il-1*β) and protein degradation markers (*Bag1* and *Bag3*) were quantified by real-time PCR. Protein levels of LC3, BAG1, and BAG3 were analyzed by immunoblotting. Real-time PCR reveals minor changes in inflammatory marker gene expression and reduced *Bag3* mRNA levels 5 days after trauma. Immunoblotting of autophagy markers LC3, BAG1, and BAG3 does not show any difference between KO and WT 24 h and 5 days after TBI. In conclusion, genetic ablation of SQSTM1-dependent autophagy leads to a delay but shows no persistent effect on post-traumatic brain damage formation. SQSTM1 therefore only plays a minor role for secondary brain damage formation and autophagic clearance of debris after TBI.

## Introduction

Immediately after impact, a complex series of biochemical events is initiated by traumatic brain injury (TBI) resulting in expansion of the primary lesion to cause secondary injury (Giza and Hovda, 2001). Destruction of brain tissue leads to an accumulation of dysfunctional organelles and protein debris in the lesion core and perilesional area, which alters functional integrity and triggers cerebral inflammation (Chen et al., 2013). Clearance of protein debris and restoration of homeostasis is of central importance to maintain normal integrity and function of the brain (Mochida et al., 2015). Autophagy (macroautophagy) is a sequestration system which facilitates fast identification and degradation of dysfunctional protein aggregates (Levine and Kroemer, 2008) and takes part in multiple physiological functions (e.g., programmed cell death and repair mechanisms) (Kuusisto et al., 2002; Mizushima et al., 2008; Weidberg et al., 2010). Degraded protein complexes are identified by receptors (co-chaperons) and are encapsulated in double membrane vesicles (autophagosomes). Autophagosomes fuse with lysosomes forming the autophagolysosomes for final degradation.

Sequestosome 1 (SQSTM1), also known as p62, is an ubiquitin-binding scaffold protein, which recognizes ubiqinated degradation prone substrates. It is one of the specific substrates degraded by the autophagic pathway. During autophagy, SQSTM1 localizes to autophagosomes by binding to microtubule-associated light chain 3 (LC3) and promoting selective autophagy of proteins in e.g., aged and acutely stressed neurons (Ichimura et al., 2008; Gamerdinger et al., 2009; Lamark et al., 2009; Rusten and Stenmark, 2010). After formation of the autolysosome, LC3 disassociates, whereas SQSTM1 and attached ubiquitin shuttles are degraded by lysosomal hydrolases (Klionsky and Emr, 2000). Importantly, SQSTM1 is a known marker for autophagic flux activity. In pathological settings like aging and disease, SQSTM1 accumulates as result of failed autophagic clearance (Moscat et al., 2016; Evans et al., 2017). Stimulation of autophagy flux causes degradation of SQSTM1 (Sarkar et al., 2014). In posttraumatic tissue signs of impaired autophagy have been observed, but molecular mechanism and consequences for brain damage formation remain controversial (Luo et al., 2011; Lipinski et al., 2015). Autophagy may be an important pro-survival factor by enabling brain tissue to overcome protein debris load. On the other hand, autophagy may serve as pro-death factor by clearing essential or required proteins involved in pro-survival cascades.

The present study investigates if an uncoupling of the SQSTM1 dependent drive of autophagy by use of SQSTM1 deficient animals is important for the formation of brain damage and impairment of neurological function after experimental TBI.

## Materials and methods

### Experimental animals

After approval by the Landesuntersuchungsamt Rheinland-Pfalz (protocol number: G14-1-037 and G12-1-010) experiments were performed in compliance with the institutional guidelines of the Johannes Gutenberg University, Mainz and in accordance with the German law for animal protection. Homozygous p62/SQSTM1 (Sqstm1^tm1a(EUCOMM)Wtsi^) mutant mice (SQSTM1^−/−^) with a neomycin cassette insertion on chromosome 11 on a C57/BL6 background and corresponding wildtype littermates (SQSTM^+/+^) were investigated (Komatsu et al., 2007). Animals were kept under standard conditions of temperature and light with free access to water and food. The food that the animals received was a standard commercial regular rodent diet.

### Experimental TBI

Under anesthesia with isoflurane (induction: 4 Vol%, maintenance: 2 Vol%) in an air mixture (40% O_2_ and 60% N_2_) via face mask, moderate focal mechanical TBI was performed by controlled cortical impact (CCI) on the right brain cortex as described previously (Sebastiani et al., 2015). Following placement into a stereotactic frame a craniotomy was performed to remove a part of the scull. A localized contusive impact was delivered to the exposed dura by a custom fabricated pneumatic controlled impactor (L. Kopacz, Mainz, Germany) which was placed perpendicular to the surface of the brain. The following variables were applied: tip diameter of 3 mm, brain penetration of 1.0 mm, impact duration of 150 ms, and impact velocity of 8 m/s. After trauma, the craniotomy was immediately sealed and the wounds were sutured closed. During surgical preparation, body temperature was maintained at 37°C by a heating plate, autoregulated, and monitored by a rectal thermocouple probe (Hugo Sachs, March-Hugstetten, Germany). Animals were returned to their own cages and placed in an incubator (33°C, 35% humidity; IC8000, Draeger, Germany) for 2 h.

### Histological evaluation of brain damage

Animals were euthanized in deep isoflurane anesthesia (4 Vol% for 1 min). Brains were quickly removed, frozen in powdered dried ice, and stored at −20°C. Each brain was cut in the coronal plane using a cryostat (HM 560 Cryo Star, Thermo Fisher Scientific, Walldorf, Germany). Sections (10 μm) were serially collected at 500 μm intervals and stained with cresyl violet according to the manufacturer's instruction. Areas of both hemispheres and the injured brain tissue were measured using a computerized image system (Delta Pix Insight, Delta Pix, Maalov, Denmark) by an investigator blinded to the randomization. Lesion volumes were calculated by multiplying contusion areas obtained from 16 consecutive sections with the distance-interval of 500 μm (0.5 ^*^ [A1 +A2 +A3 +…+ An]).

### Motor function

Motor function was analyzed by the rotarod test as described previously by an investigator blinded to the group allocation (Onyszchuk et al., 2008; Sebastiani et al., 2015). Mice were tested before, 1 and 5 days after TBI. The latency to balance until fall from the rod was recorded using a five-lane rotarod device (Panlab Rota Rod, Harvard Apparatus, Holliston, MA). For the acceleration, the speed was linearly increased from 4 to 40 rpm over 5 min. The investigation ended when the mice fell off the rod. Four rotarod tests were performed before TBI to score the baseline latencies for each animal. The average of these trials was taken as the baseline. After injury, animals were tested in two averaged trials per investigated time point.

### Neurological severity score

Before and after trauma neurological outcome was tested by an investigator blinded to the group allocation by a neurological severity adapted from Tsenter et al. (2008) which consists of 10 different tasks. These tasks are evaluating the alertness, motor ability, balancing, and general behavior of mice. Healthy mice were successful in all tasks and received 0 points. For failure to successfully perform a task 1–3 points were awarded (see Table 1).

**Table 1:** Tasks of the adapted neurological severity score.

**Task**		**Points**
Presence of a mono- or hemiparesis		1
Failure to walk a straight line		1
Startle behavior		1
Seeking behavior		1
Balance on a 1 cm-wide beam		1
Balance on a 0.5 cm-round beam		1
Exit a 25 cm-diameter wide circle	30–60 s	1
	60 s–2 min	2
	>2 min	3
Walk on a 3 cm-wide beam	>3 feet misplacement	1
	Inability to move	2
Walk on a 2 cm-wide beam	>3 feet misplacement	1
	Inability to move	2
Walk on a 1 cm-wide beam	>3 feet misplacement	1
	Inability to move	2
Maximum total		15

### RNA extraction and real-time polymerase chain reaction

Tissue preparations were performed as follows: For the time series analysis, brains were removed and placed into a cooled brain matrix (Zivic Instruments, Pittsburgh, PA). Perilesional brain tissue was dissected and immediately frozen in liquid nitrogen. For the 24-h and the 5-day studies perilesional brain tissue was collected during the cryosectioning process. Tissue was stored at −80°C until RNA isolation. Samples were homogenized in QIAzol® reagent (Qiagen). RNA isolation was performed with RNeasy® Lipid Tissue Mini kit (Qiagen) according to manufacturer's instructions. Absolute copy numbers of target genes were normalized against the housekeeping gene cyclophilin A (PPIA) (Thal et al., 2008). For applied primer sequences see Table 2. Same amounts of cDNA were amplified in duplicates using Absolute Blue qPCR SYBR Green Mix (Thermo fisher Scientific) for *Ppia, Tnf-*α*, inos*, and *Sqstm1*, Maxima Probe qPCR Mastermix (Thermo Fisher Scientific) for *Il-1*β and *Bag3*, Light Cycler 480 Probes Master (Roche) for *Bag1*, and Quanti Nova qPCR kit (Qiagen) for *IL-6* according to the manufacturer's instructions.

**Table 2:** Specific primer and probes and optimized temperature conditions for real-time polymerase chain reaction (PCR, polymerase chain reaction; Forw, sense primer; Rev, antisense primer; Cy5, Cyanine 5; Phos, Phosphate; FL, fluorescein).

**PCR assay (amplicon size, annealing temp)**	**Oligonucleotide Sequence (5′–3′)**	**Gene bank no**.
Cyclophilin A (PPIA) (146 bp, 55°C)	Forw: 5′-GCGTCTSCTTCGAGCTGTT-3′ Rev: 5′-RAAGTCACCACCCTGGCA-3′ FL: 5′-GCTCTGAGCACTGGRGAGAAAGGA-FL Cy5: Cy5-TTGGCTATAAGGGTTCCTCCTTTCACAG-Phos	NM_008907
Bag1 (306 bp, 55°C)	Forw: 5′-CACCCACAGCAATGAGAGGTAT-3′ Rev: 5′-AATTCTTGCAGGTGGTTAGCTATC-3′ FL: 5′-GGGAAAATCTCTGAAAGAAATGGAAACACC-FL Cy5: Cy5-TTGTCAGCACTTGGAATGCAAAATGGT-Phos	NM_001171739
Bag3 (269 bp, °C)	Forw: 5′-CACCACGACGTGGAACG-3′ Rev: 5′-GGGACCTCTGCGGAGTG-3′ FL: 5′-GCTCCGACCAGGCTACATTCCCA-FL Cy5: Cy5-CCCCGTCCTCCATGAAGGCTCCG-Phos	NM_013863
Sqstm1 (292 bp, 55°C)	Forw: 5′-CTCAGCCCTCTAGGCATTGAG-3′ Rev: 5′-AAGACAAATGTGTCCAGTCATCGT-3′ FL: 5′-CCTTGGAGTCGGTGGGACAGCC-FL Cy5: Cy5-GAACAGATGGAGTCGGGAAACTGCTCA-Phos	NM_011018
IL-1β (348 bp, 55°C)	Forw: 5′-59-GTGCTGTCGGACCCATATGAG-3′ Rev: 5′-CAGGAAGACAGGCTTGTGCTC-3′ FL: 5′-TAATGAAAGACGGCACACCCACCC-FL Cy5: Cy5-CAGCTGGAGAGTGTGGATCCCAAGC-Phos	NM_008361
IL-6 (141 bp, 55°C)	Forw: 5′-GAGGATACCACTCCCAACAGACC-3′ Rev: 5′-AAGTGCATCATCGTTGTTCATACA	NM_031168
TNF-α (212 bp, 62°C)	Forw: 5′-TCTCATCAGTTCTATGGCCC-3′ Rev: 5′-GGGAGTAGACAAGGTACAAC-3′	NM_ 008361
iNOS (NOS2) (312 bp, 55°C)	Forw: 5′-TGTGTCAGCCCTCAGAGTAC-3′ Rev: 5′-CACTGACACTYCGCACAA-3′ R640: Red-GCTCCTCCCAGGACCACACCC-Phos FL: 5′-GAAGCCCCGCTACTACTCCATC-FL	NM_010927

### Immunoblotting

Perilesional brain tissue was homogenized in radioimmunoprecipitation assay buffer (50 mM Tris-HCl, pH 7.4; 150 mM NaCl; 0.5 mM EDTA; 0.5% NP-40; protease and phosphatase inhibitors). Protein concentration was determined by Lowry Protein Assay (Promega, Madison, WI, USA) and 40–50 μg of each sample were separated in 12%-SDS polyalcryamide gel electrophoresis (SDS-PAGE), for spectrin and SQSTM1 in 4–12% NuPAGE (4–12% Bis-Tris Protein Gel, 1.5 mm; Thermofisher Scientific, Waltham, MA, USA), respectively, and transferred to a nitrocellulose membrane. After blocking with 5% skimmed milk for 1.5 h at room temperature and washing with Tris-buffered saline with 1% Tween-20 (Carl Roth, Karlsruhe, Germany) primary antibodies against SQSTM1 (1:1,000; GP62-C, Progen, Heidelberg, Germany), BAG3 (1:500, 10599-1-AP, Proteintech, Rosemont, IL, USA), BAG1 (1:500, aliquot kindly provided by Prof. Dr. Franz-Ulrich Hartl, Max-Planck-Institute of Biochemistry, Martinsried, Germany), spectrin (1:750, BML-FG6090, Enzo Life Science, Farmingdale, NY, USA), and LC3 (1:1,000, NB100-2220, Novus Biologicals, Littleton, CO, USA) or GAPDH (1:4,000, ACR001PS, Acris, Rockville, MD, USA) as loading control were applied in TBST or 2.5% skimmed milk (BAG3, Spectrin, GAPDH) and incubated overnight at 4°C, respectively, 2 h at room temperature (GAPDH). Secondary Antibodies against mouse, rabbit or guinea-pig (Li-Cor Bioscience, Lincoln, NE, USA) were applied for 1 h at room temperature. After washing protein bands were detected using Odyssey Imaging Systems and their intensities were measured with Image Studio Version 3.1 (both Li-Cor Bioscience, Lincoln, NE, USA) and normalized to the sample's GAPDH expression.

### Statistical analysis

All experiments were randomized and performed by an investigator blinded to the group allocation. Analysis was performed by Sigma Plot 12.5 software (Systat Software Inc., San Jose, CA, USA). Exact Wilcoxon Mann–Whitney tests were used and values were adjusted for multiple comparisons with the Holm–Bonferroni method. *P* < 0.05 was considered to be significant. Graph bars indicate mean and standard deviation throughout the figures.

## Results

### *S*QSTM1 is reduced after experimental TBI

**Figure 1:**
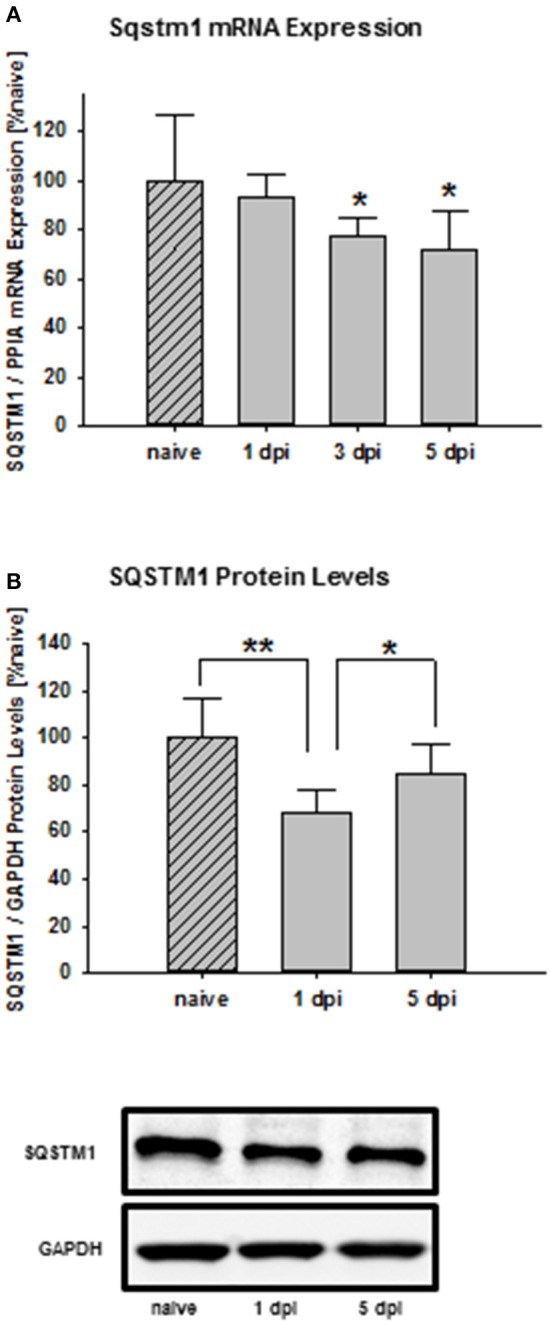
*SQSTM1* is reduced after TBI. Time course of the posttraumatic *Sqstm1* mRNA regulation in perilesional cortical brain tissue was measured by real-time PCR **(A)**. Additionally, protein levels of SQSTM1 were measured by western blot analysis in perilesional tissue lysates **(B)**. The results are presented at different recovery time points after trauma. *Sqstm1* mRNA expression and SQSTM1 protein levels are down-regulated following trauma. *Sqstm1*, sequestosome 1; dpi, day(s) post injury; data are expressed as mean ± *S.D*.; *P* values are adjusted for multiple comparisons; ^*^P < 0.05; ^**^P < 0.01.

Time frame analysis shows that *Sqstm1* mRNA expression is suppressed 3 days after trauma compared with naïve mice and stays depressed until day 5 after injury (naïve: 100.0 ± 26.8% naïve; 1 day post injury: 93.7 ± 8.9% naïve; *P* = 0.566 vs. naïve; 3 days post injury: 77.4 ± 7.4% naïve, *P* = 0.0377 vs. naïve; 5 days post injury: 71.0 ± 16.3% naïve; *P* = 0.0311; *n* = 9–10 mice/group; Figure 1A). Additionally, SQSTM1 protein levels were investigated by immunoblotting. SQSTM1 protein levels are reduced 1 day after TBI and nearly return to naïve values 5 days after trauma (naïve: 100.0 ± 17.2% naïve; 1 day post injury: 68.1 ± 9.7% naïve, *P* = 0.0071 vs. naïve; 5 days post injury: 85.0 ± 12.2, *P* = 0.042 vs. 1 day post injury; *n* = 5 mice/group; Figure 1B).

### The lack of SQSTM1 delays formation of brain damage

**Figure 2:**
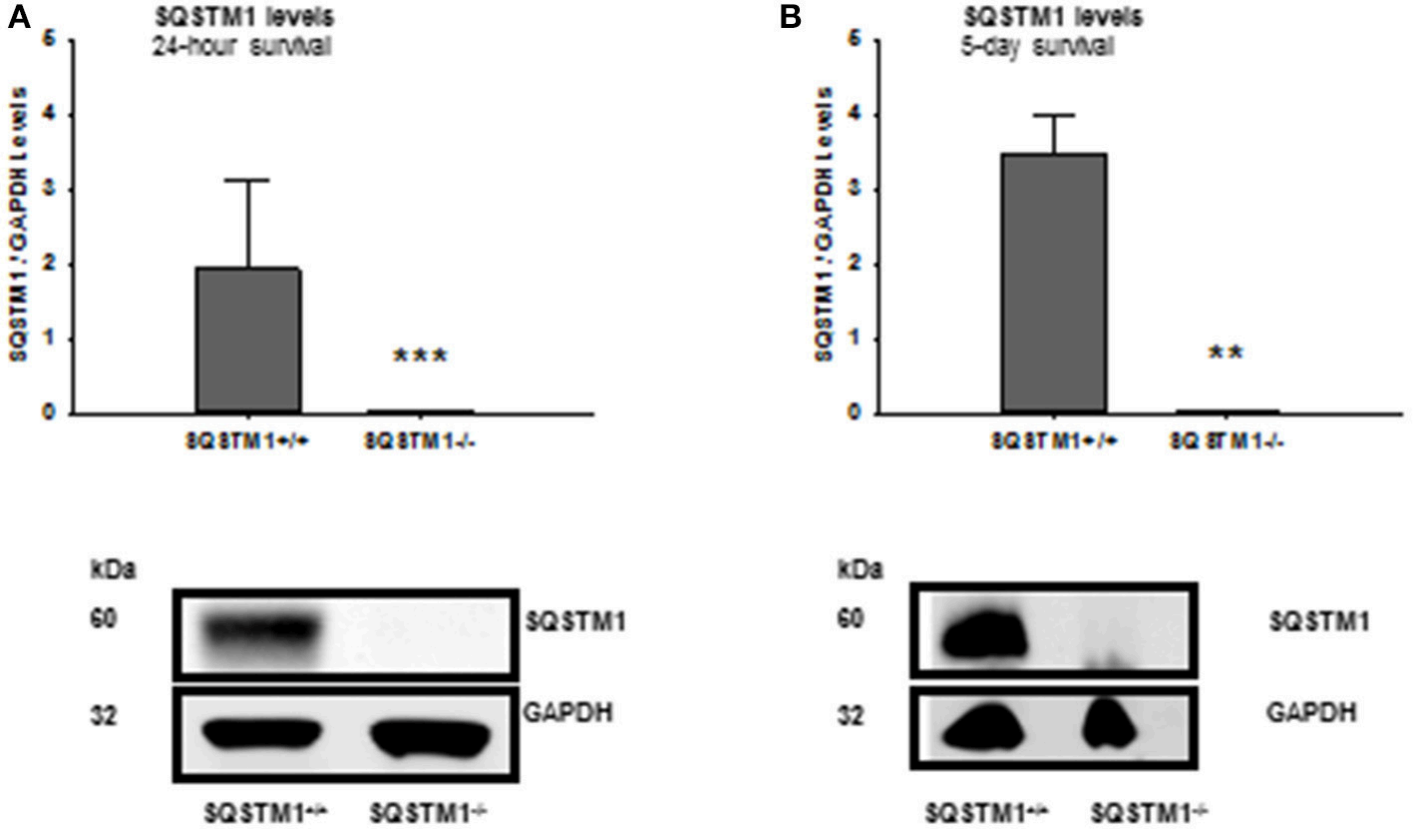
Confirmation of SQSTM1 knockout animals. The absence of SQSTM1 of investigated animals is confirmed by western blot analysis of brain tissue lysates 24 h and 5 days after CCI. SQSTM1, sequestosome 1; GAPDH, glyceraldehyde-3-phosphate dehydrogenase, data are expressed as mean ± *S.D*.; ^**^P < 0.01; ^***^P < 0.001.

**Figure 3:**
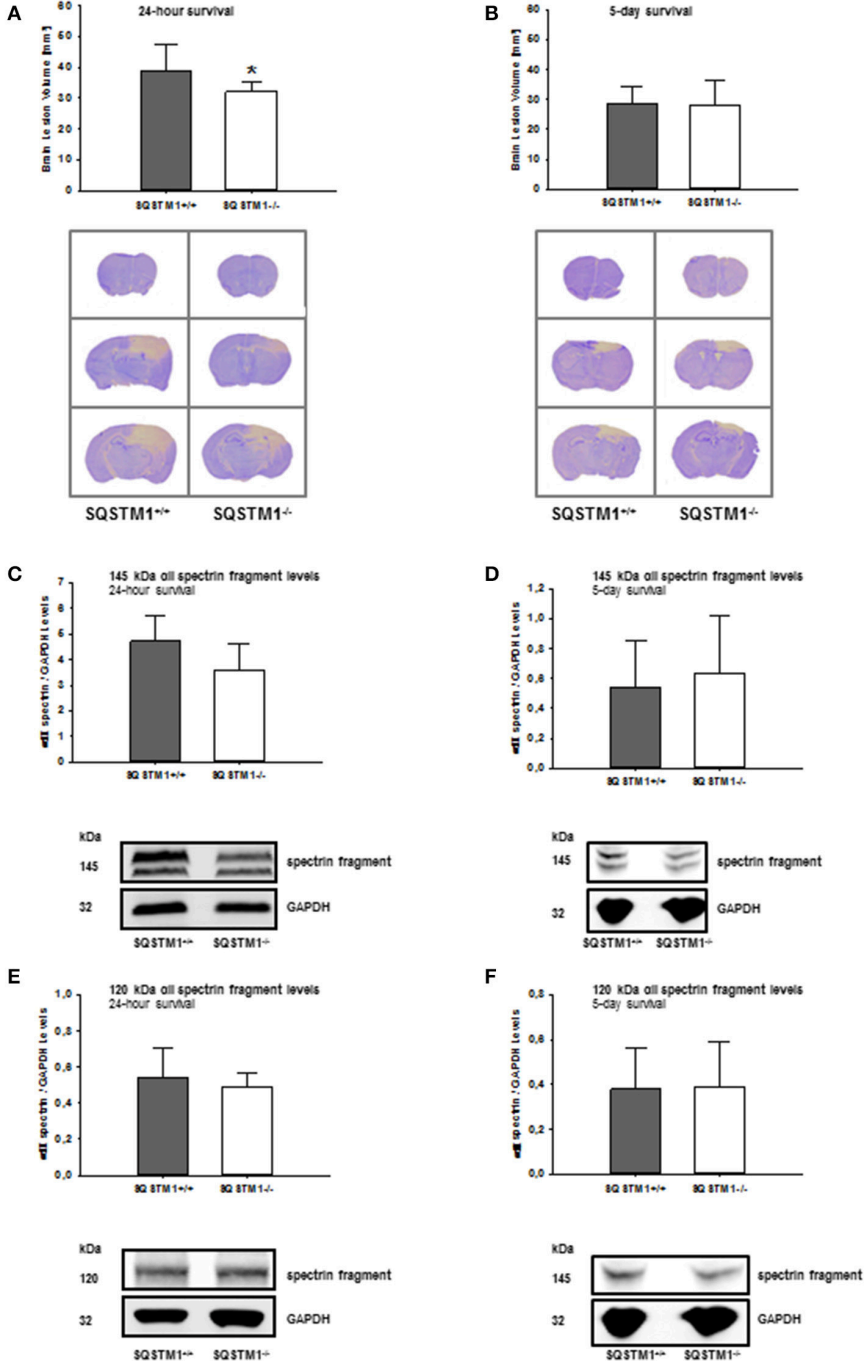
SQSTM1 plays a role in early secondary brain damage formation after TBI. Lesion volume of SQSTM1^−/−^ mice is reduced compared with SQSTM1^+/+^ littermates 24 h after experimental TBI **(A)**. 5 days after trauma there is no difference between SQSTM1^−/−^ and SQSTM1^+/+^ mice. Representative cresyl-violet stained sections at the coronal plane from 1.70 mm anterior to bregma, 0.46 mm posterior to bregma, and 1.46 mm posterior to bregma at 24 h **(A)** and 5 days **(B)** after CCI are shown (according to The Mouse Brain Library: www.mbl.org). Protein analysis was performed by immunoblotting to determine 120 and 145 kDa αII-spectrin fragments in perilesional brain tissue samples. In SQSTM1^−/−^ mice calpain-dependent spectrin proteolysis to 145/150-kDa fragments is reduced by trend 24 h after CCI **(C)**. There is no effect on calpain-dependent cell death 5 days after CCI **(D)**. SQSTM1^−/−^ does not influence caspase-dependent spectrin proteolysis to 120-kDa fragments **(E,F)**. SQSTM1, sequestosome 1; GAPDH, glyceraldehyde-3-phosphate dehydrogenase, data are expressed as mean ± *S.D*. ^*^P < 0.05.

SQSTM1 knockout is confirmed by western blot analysis in brain tissue lysates of investigated animals 24 h (*P* < 0.001; *n* = 7/group; Figure 2A) and 5 days after trauma (*P* = 0.008; *n* = 5/group; Figure 2B). Twenty four hours following trauma lesion volume is smaller in SQSTM1^−/−^ (32.1 ± 3.5 mm^3^, *n* = 8 mice/group) compared with SQSTM1^+/+^ mice (38.8 ± 8.6 mm^3^, *n* = 12; *P* = 0.025; Figure 3A). Additionally, lesion volume was analyzed 5 days after experimental TBI. Lesion volumes in SQSTM1^−/−^ and wildtype littermates are indistinguishable (28.8 ± 5.8 vs. 28.3 ± 8.0 mm^3^; *P* = 0.937; *n* = 6 mice/group; Figure 3B). To determine the influence of SQSTM1 absence on neuronal cell death, perilesional brain samples of SQSTM1^−/−^ and SQSTM1^+/+^ mice were investigated 24 h and 5 days after trauma. Western blot analysis of caspase-dependent 120 kDa αII-spectrin fragment and calpain-dependent 145 kDa αII-spectrin was performed. Calpain-dependent cell death is reduced by trend in SQSTM1^−/−^ mice compared with SQSTM1^+/+^ littermates 24 h after injury (*P* = 0.0797; *n* = 7 mice/group; Figure 3C). In line with the data investigating brain damage formation, there is no difference in 145 kDa αII-spectrin fragment levels 5 days after trauma between SQSTM1^−/−^ and SQSTM1^+/+^ animals (*P* = 0.6633; *n* = 5 mice/group, Figure 3D). SQSTM1 absence does not influence caspase-dependent αII-spectrin cleavage 24 h (*P* = 0.507; *n* = 7 mice/group, Figure 3E) or 5 days after TBI (*P* = 0.9339; *n* = 5 mice/group, Figure 3F).

### Neurological function is not affected by SQSTM1 mutation

**Figure 4:**
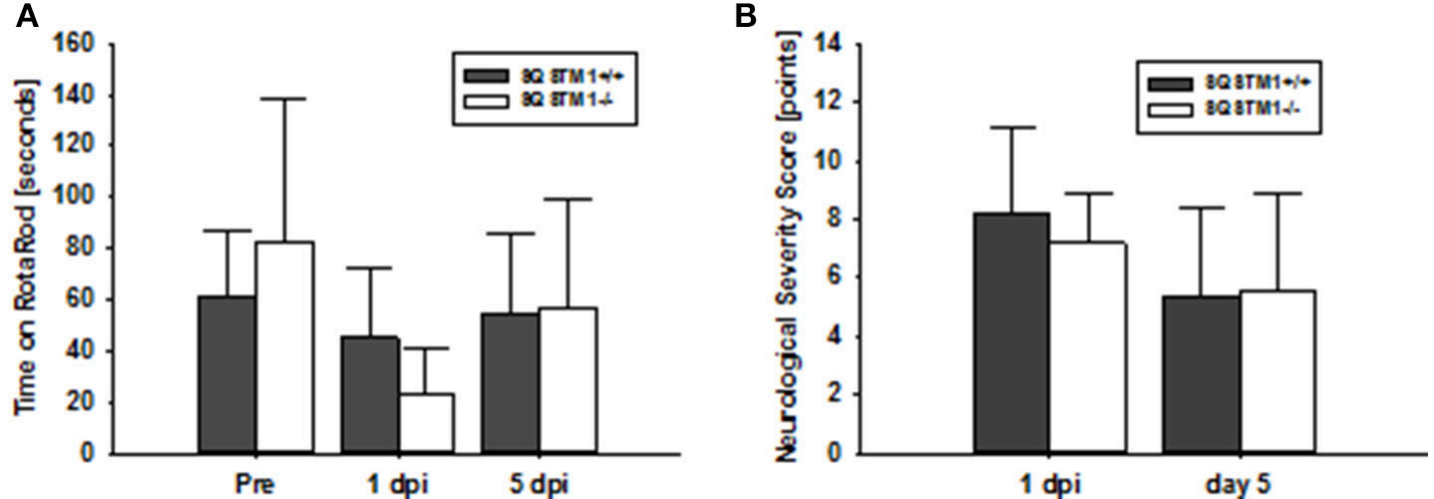
SQSTM1 does not influence neurological function after TBI. Neurological function was investigated by RotaRod analysis **(A)** and a standardized neurological severity score **(B)**. Both tests reveal no difference between SQSTM1^−/−^ and SQSTM1^+/+^ littermates. Pre, before trauma; dpi, day(s) post injury; data are expressed as mean ± *S.D*.

Motor coordination was analyzed by rotarod before, 24 h and 5 days after trauma in SQSTM1^−/−^ and corresponding SQSTM1^+/+^ littermates. SQSTM1 deficiency does not influence motor coordination compared with littermate animals 24 h (*P* = 0.065) or 5 days (*P* = 0.818) after TBI (*n* = 6 mice/group; Figure 4A). Neurological Severity Score does not reveal any differences between SQSTM1^−/−^ and SQSTM1^+/+^ 24 h (*P* = 0.699) or 5 days (*P* = 0.937) after trauma (SQSTM1^+/+^, 24 h: 8.2 ± 3.0 points, 5 days: 5.3 ± 3.1 points; SQSTM1^−/−^, 24 h: 7.2 ± 1.7 points, 5 days: 5.5 ± 3.4 points, *n* = 6 mice/group; Figure 4B).

### SQSTM1 deficiency influences *Tnf-α* and *Il-6* mRNA expression after TBI

**Figure 5:**
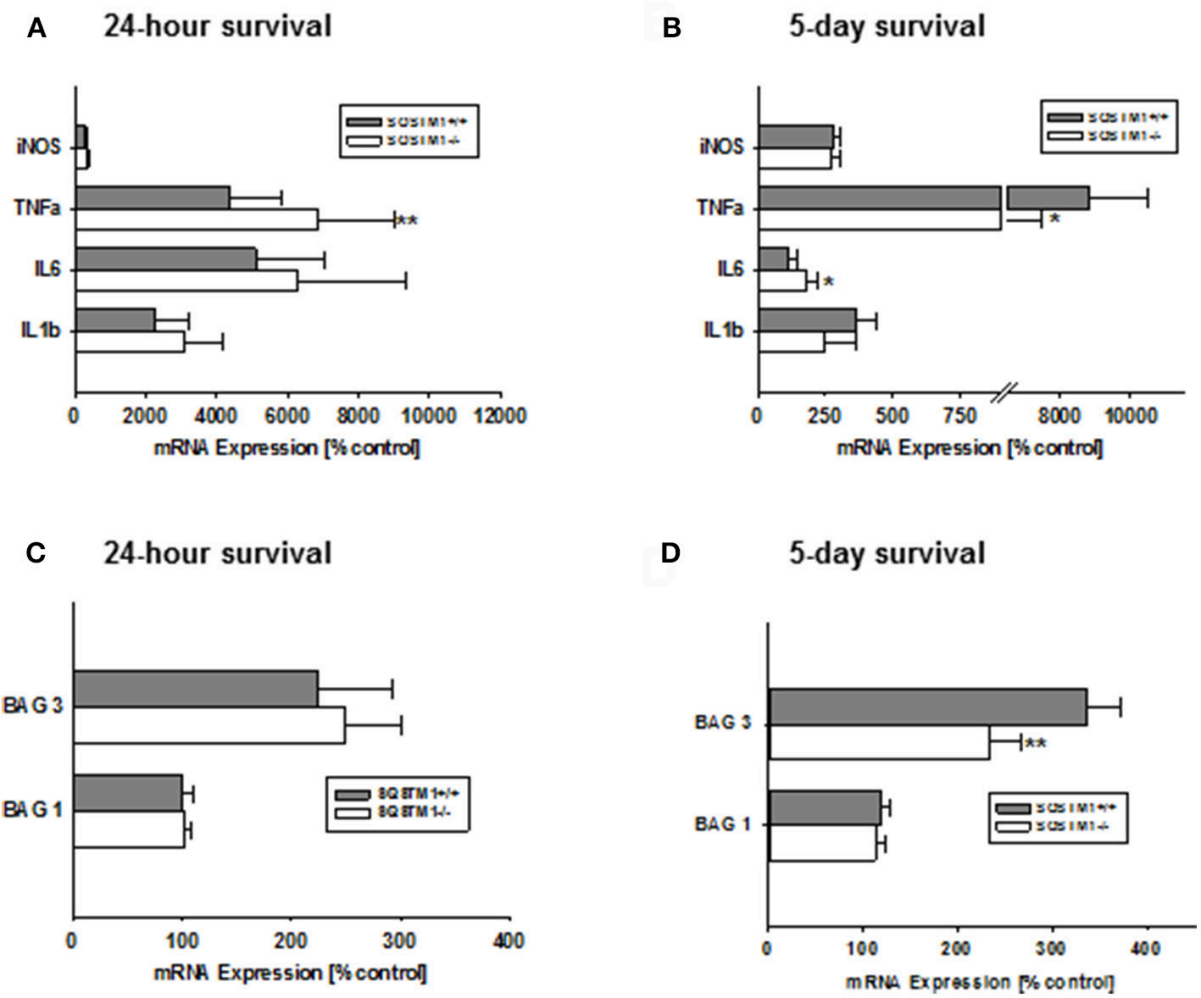
Effect of SQSTM1 deficiency on inflammatory and protein degradation marker genes. SQSTM1^−/−^ mice show increased *Tnf-*α expression levels 24 h after trauma. Other investigated inflammatory marker genes are not changed 24 h following trauma **(A)**. 5 days after trauma *Tnf-*α expression levels are decreased and *Il-6* expression is increased in SQSTM1^−/−^ mice. There are no changes in the other investigated inflammatory marker genes 5 days after trauma **(B)**. Bag 1 mRNA expression is not influenced by SQSTM1^−/−^ **(C)**. 5 days after trauma *Bag3* expression is reduced in SQSTM1^−/−^ **(D)**. *inos*, Inducible nitric oxide synthase; *Tnf-*α, tumor necrosis factor-α; *Il-6*, interleukin-6; *Il-1*β, interleukin-1β; *Bag1*, BCL-2 associated athanogene 1; *Bag3, BCL-2* associated athanogene 3; *Sqstm1*, sequestosome 1; data are expressed as mean ± *S.D*.; *P* values are adjusted for multiple comparisons; ^*^P < 0.05; ^**^P < 0.01.

Gene expression levels of the pro-inflammatory markers *iNos, Tnf-*α*, Il-6*, and *Il-1*β were investigated 24 h and 5 days after trauma by real-time PCR. SQSTM1 deficiency increases inflammatory marker *Tnf-*α expression 24 h after head trauma (SQSTM1^−/−^: 4,378 ± 1,424% control; SQSTM1^+/+^: 6,820 ± 2,186% control, *P* = 0.005; *n* = 10–12 mice/group; Figure 5A). Five days after head trauma *Tnf-*α levels are lower in SQSTM1 deficient animals (SQSTM1^−/−^: 5,976 ± 1,501% control; SQSTM1^+/+^: 8,837 ± 1,643% control, *P* = 0.026; *n* = 6 mice/group; Figure 5B), whereas *Il-6* expression levels are significantly higher in SQSTM1^−/−^ animals at 5 days after insult (SQSTM1^−/−^: 179 ± 46% control; SQSTM1^+/+^: 115 ± 29% control, *P* = 0.015; *n* = 6 mice/group, Figure 5B). There is no difference between SQSTM1^−/−^ and corresponding SQSTM1^+/+^ in the expression levels of other investigated markers such as *iNos* (24 h: SQSTM1^−/−^: 323 ± 82% control; SQSTM1^+/+^: 281 ± 75% control, *P* = 0.221; *n* = 10–12 mice/group; 5 days: SQSTM1^−/−^: 271 ± 37% control; SQSTM1^+/+^: 284 ± 23% control, *P* = 0.310; *n* = 6 mice/group), *Il-6* (24 h: SQSTM1^−/−^: 6,250 ± 3,090% control; SQSTM1^+/+^: 5,108 ± 1,921% control, *P* = 0.646; *n* = 10–12 mice/group), and *Il-1*β (24 h: SQSTM1^−/−^: 3,075 ± 1,075% control; SQSTM1^+/+^: 2,282 ± 909% control, *P* = 0.061; *n* = 10–12 mice/group; 5 days: SQSTM1^−/−^: 249 ± 114% control; SQSTM1^+/+^: 368 ± 69 % control, *P* = 0.093; *n* = 6 mice/group; Figures 5A,B).

### SQSTM1 deficient animals show lower *Bag3*, but unchanged *Bag1* mRNA expression levels

In order to investigate if the potential mechanism of the early effect on lesion volume in by SQSTM1^−/−^ is due to changes in key regulators of protein degradation, gene expression levels for *Bag1* (marker of proteasomal activity) and *Bag3* (mediator of the BAG3-mediated selective macro-autophagy pathway; Gamerdinger et al., 2009) were analyzed by real-time PCR. In SQSTM1^−/−^ mice *Bag3* expression levels are significantly lower 5 days after TBI (24 h: SQSTM1^−/−^: 102 ± 5% control; SQSTM1^+/+^: 100 ± 11% control, *P* = 0.589; 5 days: SQSTM1^−/−^: 234 ± 33% control; SQSTM1^+/+^: 336 ± 36% control, *P* = 0.002, Figures 5C,D). *Bag1* expression levels are not changed by SQSTM1 deficiency (24 h: SQSTM1^−/−^: 248 ± 52% control; SQSTM1^+/+^: 225 ± 68% control, *P* =; 5 days: SQSTM1^−/−^: 113 ± 10% control; SQSTM1^+/+^: 119 ± 9% control, *P* = 0.485; Figures 5C,D).

### Autophagy and proteasomal degradation is not influenced by SQSTM1 deficiency

**Figure 6:**
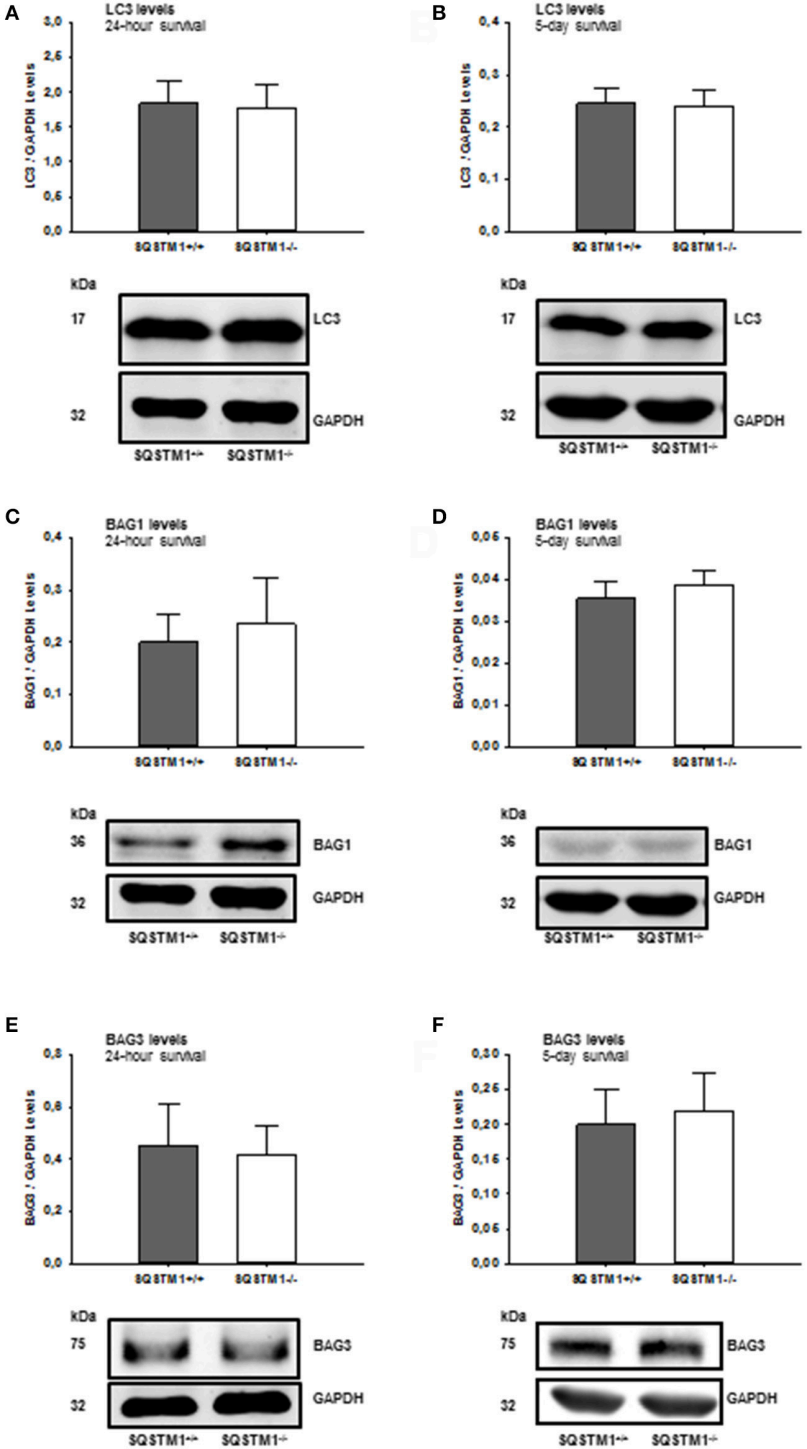
SQSTM1^−/−^ does not influence protein degradation marker levels. Protein analysis was performed by western blotting to determine LC3, BAG1, and BAG3 protein content in perilesional brain tissue samples. Immunoblotting does not reveal any difference in SQSTM1^−/−^ compared with SQSTM1^+/+^ littermates in the content of autophagy markers LC3 **(A,B)**, BAG1 **(C,D)**, and BAG1 **(E,F)** (marker of proteasomal degradation). BAG1, BCL-2 associated athanogene 1; BAG3, BCL-2 associated athanogene 3; SQSTM1, sequestosome 1; data are expressed as mean ± *S.D*.

In order to confirm the results of mRNA analysis, the impact of SQSTM1 deficiency on protein degradation marker levels was investigated by western blot of perilesional brain tissue lysates in SQSTM1^−/−^ and SQSTM1^+/+^ littermates 24 h and 5 days after trauma. Autophagy markers LC3 (24 h: *P* = 0.677; *n* = 8 mice/group; 5 days: *P* = 0.771, *n* = 6 mice/group; Figures 6A,B) and BAG3 (24 h: *P* = 0.610; *n* = 8 mice/group; 5 days: *P* = 0.561, *n* = 6 mice/group; Figures 6E,F) and proteasomal activity marker BAG1 (24 h: *P* = 0.354; *n* = 8 mice/group; 5 days: *P* = 0.230, *n* = 6 mice/group; Figures 6C,D) protein content is unchanged in the absence of SQSTM1.

## Discussion

Several studies have identified the importance of dysregulated autophagy in different human diseases. The ubiquitin-binding protein SQSTM1 was identified as key component of the selectivity of the autophagy network. The present study investigates the role of SQSTM1-mediated autophagy for brain damage formation after experimental TBI. Our study demonstrates (I) reduced *Sqstm1* mRNA expression and SQSTM1 protein levels indicating increased autophagy flux after TBI and (II) that absence of SQSTM1 leads to delayed but no long term effect on brain damage formation after TBI.

Depending on the situation autophagy may serve as pro-survival or pro-death mechanism. Autophagy is constitutively present at low levels in all healthy neurons (Rubinsztein et al., 2005). A dysregulation of this degradation pathway has been reported to occur in many neurodegenerative processes like post-traumatic stress disorder (Zheng et al., 2017), cerebral ischemia (Xu et al., 2016), Parkinson's disease (Wang et al., 2016), or Alzheimer's disease (Kuusisto et al., 2002).

The ubiquitin receptor SQSTM1 is an important player of the autophagic pathway. Recent findings suggest that SQSTM1-dependent autophagy negatively regulates important signaling pathways (Niida et al., 2010; Sandilands et al., 2012), key downstream components and transcription factors (Wang et al., 2011) changes in stress conditions (Belaid et al., 2014). It directly interacts with LC3 and is incorporated and sequestered within an autolysosome for degradation and thereby acts an autophagy substrate (Pankiv et al., 2007). Thus, SQSTM1 is used to evaluate the autophagic flux (Sahani et al., 2014; Geng and Klionsky, 2017). Vice versa, autophagy is responsible for the degradation of SQSTM1. Hence, an impairment of autophagy is usually accompanied by an accumulation of SQSTM1 protein (Komatsu and Ichimura, 2010; Katsuragi et al., 2015). In the present study *Sqstm1* mRNA expression and SQSTM1 protein levels decrease after trauma compared with the naïve group indicating an induction of autophagy flux (Ichimura et al., 2008). Decreased *Sqstm1* mRNA expression levels following insult have also been shown for other brain pathologies like hypoxic-ischemic brain injury (Xu et al., 2016). In contrast to the present data, other studies have shown accumulation of SQSTM1 following experimental TBI compared with sham animals (Sarkar et al., 2014). Consistently, in a study investigating the cerebrovascular fluid of 30 children with severe TBI, increased SQSTM1 protein levels where detected in the cerebrovascular fluid after TBI compared to a control group. In this study, peak levels of SQSTM1 were higher in patients with unfavorable outcome (Au et al., 2016).

These data suggest that modulation of the autophagy flux in SQSTM1^−/−^ mice should have an influence on pathophysiological mechanisms after acute brain trauma. Unfortunately, our results did not confirm this hypothesis and failed to show a significant influence on brain damage 5 days after trauma. At a very early time point after insult SQSTM1^−/−^ mice demonstrated a significant lower lesion volume at 24 h and by trend decreased calpain-dependent cell death after TBI compared with wildtype littermates. In line with the histological data, neurofunctional data was not significantly different between groups. Deficiency of SQSTM1 therefore delays, but does not prevent secondary brain damage. A possible explanation for this effect might be that SQSTM1 severely impairs mitochondrial function and, thereby enhances neurodegeneration (Jackson et al., 2017). SQSTM1 deficiency results in inhibition of SQSTM1-dependent autophagy and will result in accumulation of latter protein substrates. The data suggest that prevention of degradation may be a physiological reaction, as indicated by mRNA levels. Further inhibition of the pathways was able to further delay but not prevent damage evolution.

Additionally, the influence of SQSTM1^−/−^ on BAG1 and BAG3, other main participators of the protein degradation system, was investigated. Following brain trauma SQSTM1^−/−^ mice show decreased *Bag3* mRNA expression levels 5 days after trauma. Thereby, SQSTM1 deficiency might additionally counteract an autophagy overshoot by decreasing the expression of one of its main key players BAG3. In line with this, a neuroprotective effect of BAG3 depletion has been also described for neuronal hypoxia-ischemia injury. Here, BAG3 depletion prevented hippocampal neuronal death via depression of galactin 3 and filamin c (Cho et al., 2012). Protein degradation markers are unchanged in SQSTM1^−/−^ after TBI indicating its minor role in early pathophysiological events after TBI.

Several other studies have demonstrated neuroprotective effects by addressing other players of autophagy. Mice deficient in Atg7, essential for autophagy induction, showed protection from hypoxia-ischemia induced caspase-3 activation and neuronal cell death (Koike et al., 2008). In a mouse model of frontotemporal dementia, inhibition of autophagy ameliorated neuronal cell loss. The authors suggested that an excessive accumulation of autophagosomes is detrimental for neuronal survival under some neurodegenerative conditions (Lee and Gao, 2009). Our present data do not support the notion that inhibition of autophagy, specifically the SQSTM1-dependent autophagy has a substantial and lasting effect on secondary brain damage.

## Conclusion

The findings of the present study have important implications for a better understanding of SQSTM1-dependent autophagy and its involvement in cerebral trauma. The modulation of autophagy in SQSTM1^−/−^ animals causes an attenuation of cell death processes, but does not prevent secondary brain damage and fails to influence neurological function after trauma. Therefore, data suggest a minor role of SQSTM1-dependent autophagy for pathophysiological mechanisms in the initial phase after head injury.

## Author contributions

Conceived and designed the experiments: AS, CG, ST, and KE; Performed the experiments: AS, CG, PS, and WB. Analyzed the data: AS, CG, PS, WB, TM, CB, ST, and KE. Wrote the paper: AS, CG, PS, TM, ST, and KE.

### Conflict of interest statement

The authors declare that the research was conducted in the absence of any commercial or financial relationships that could be construed as a potential conflict of interest.
